# Supplementary figures and images for: Quality of life disparities among Mexican people with systemic lupus erythematosus
Source: PLOS Digit Health. 2025 Jan 23;4(1):e0000706. doi: 10.1371/journal.pdig.0000706 (PMC11756751; doi:10.1371/journal.pdig.0000706)

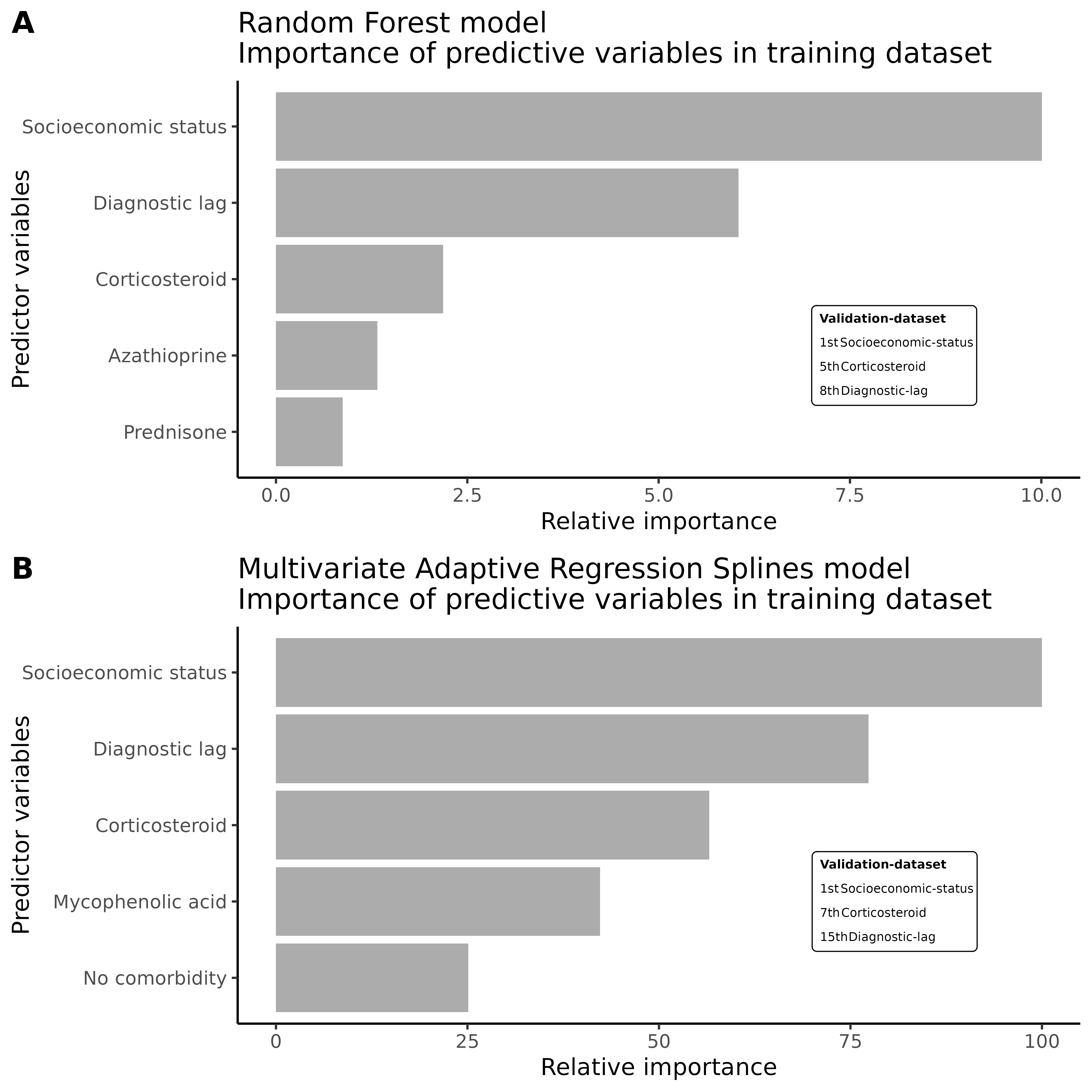

Supplement: S1 Fig — (A) Random forest model and (B) multivariate adaptative regression splines model. (TIF) [file pdig.0000706.s001.tif]

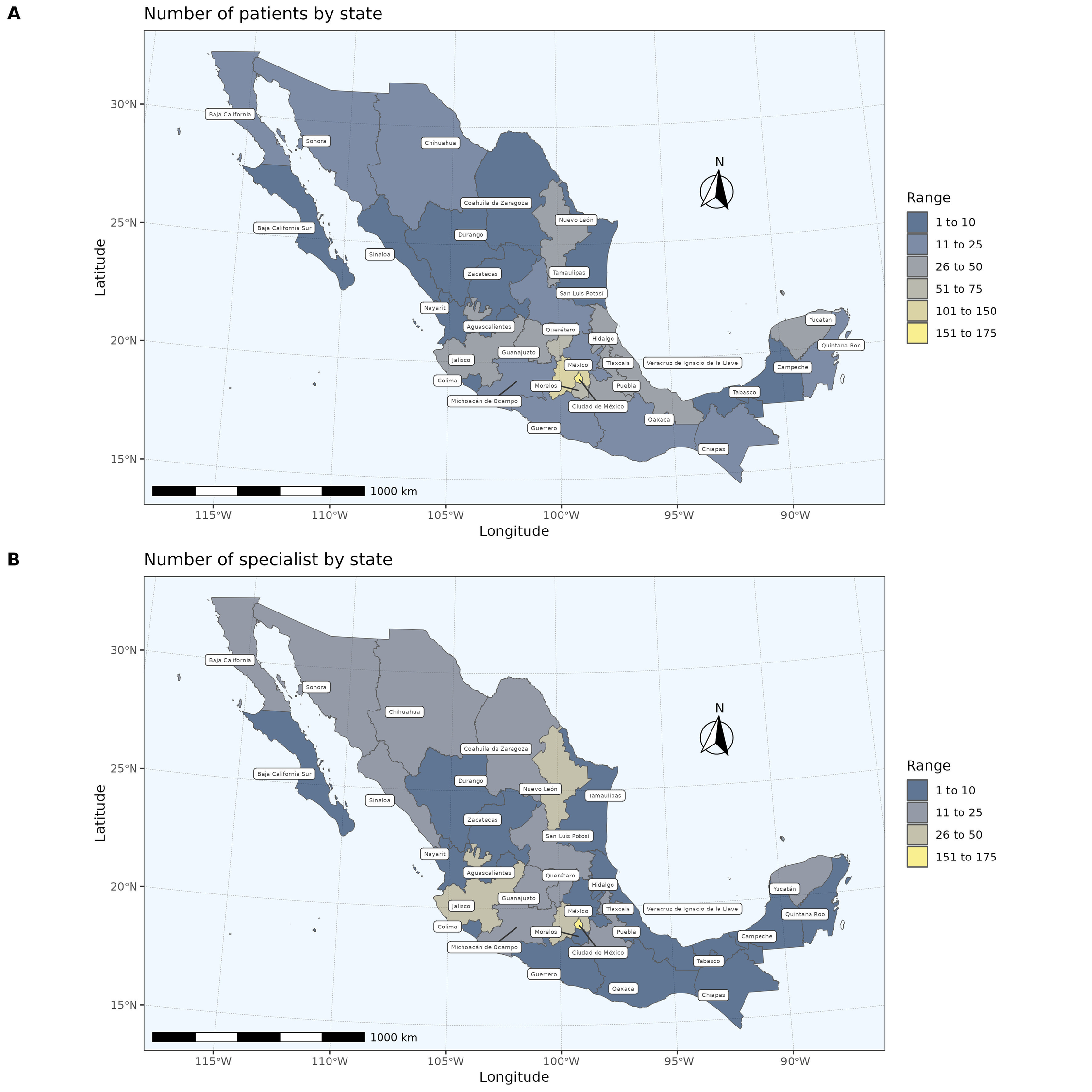

Supplement: S2 Fig — (A) Number of patients by state, and (B) number of specialists (rheumatologists) by state. Map created in R, with source shape files from Instituto Nacional de Estadistica y Geografia (INEGI). (2022). Marco Geoestadistico Estados Unidos Mexicanos. Recovered from [https://www.inegi.org.mx/app/biblioteca/ficha.html?upc=889463770541]. Terms of use: [https://www.inegi.org.mx/inegi/terminos.html]. (TIF) [file pdig.0000706.s002.tif]
