# Supplementary material for: Quality of life disparities among Mexican people with systemic lupus erythematosus
Source: PLOS Digit Health. 2025 Jan 23;4(1):e0000706. doi: 10.1371/journal.pdig.0000706 (PMC11756751; doi:10.1371/journal.pdig.0000706)
Supplement: Attachment — Submitted filename: comments.pdf [file pdig.0000706.s003.pdf]

No	Questions	
1	Lines 172-178	Why were participants only recruited through social media and community outreach? How does this recruitment strategy impact the representativeness of the sample?
2	Lines 186-190	How was the validity of the self-reported SLE diagnosis confirmed in the absence of medical records?
3	Lines 199-208	Can you explain how socioeconomic status was quantified and how this impacts the findings?
4	Lines 214-223	What statistical methods were used to handle missing data and potential biases in the dataset?
5	Lines 240-245	How did you address the potential confounders in the random forest model?
6	Lines 255-261	Were there any efforts to blind the data analysts to the study hypothesis to reduce bias?
7	Lines 300-310	What measures were taken to ensure that the control groups were adequately matched to the SLE patients?
8	Lines 320-325	How does the diagnostic lag affect the management and prognosis of SLE in different socioeconomic groups?
9	Lines 413-425	Could you discuss further the potential biases introduced by the high female-to-male ratio in your sample?
10	Lines 199-208	How did you ensure that the survey instruments were culturally and linguistically appropriate for the diverse Mexican population?

Analysis	214-278	Provide more detailed explanations for the choice of specific parameters in the models, such as the hyperparameters in the random forest model. Justify the use of non-parametric tests and the treatment of Likert scale data as numeric variables.
Literature	General	Discuss more recent studies focusing on the Mexican population or similar contexts. Provide a broader range of studies from different geographical regions.
	122-163 (Introduction)	Include more recent references in the introduction. Expand the range of studies discussed to provide a more comprehensive overview.
Methodology	164-213	Include information on the validation of the questionnaires used in the Mexican context. Further elaborate on the rationale behind choosing specific statistical tests and models.
	214-278 (Statistical analysis)	Provide a more detailed rationale for the choice of statistical methods and parameters. Discuss limitations or assumptions of the statistical methods used.
Discussion	345-451	Discuss the limitations of the study more thoroughly, including potential biases in self-reported data and the representativeness of the sample. Provide more specific recommendations for policy or clinical practice based on the findings to make the conclusions more actionable. Enhance the discussion by linking the findings more explicitly to the existing literature and providing a broader context for the study's implications.

No	Suggestions for Improvement	
1	Lines 172-178	Consider expanding the recruitment strategy to include clinical settings to ensure a more representative sample of the general SLE population, potentially increasing the generalizability of the findings.
2	Lines 186-190	Implement a validation study to compare self-reported data with medical records for a subset of participants to enhance the reliability of the diagnostic information.
3	Lines 199-208	Provide a more detailed explanation of the socioeconomic scoring system used and its validation process to help readers understand its application and impact on the study results.
4	Lines 214-223	Describe the methods used for imputing missing data and discuss the sensitivity analyses conducted to assess the impact of these methods on the study outcomes.
5	Lines 240-245	Clearly define all confounders considered in the study and describe the statistical techniques used to control for these in the modeling process.
6	Lines 255-261	Introduce blinding techniques where possible during the analysis phase to minimize bias in the interpretation of the data.
7	Lines 300-310	Ensure that control groups are recruited from similar backgrounds as the SLE patients, using stratification or matching criteria based on key demographic and clinical characteristics.
8	Lines 320-325	Explore the implications of diagnostic delays in more depth, particularly how they differ across socioeconomic statuses and what interventions could mitigate these disparities.
9	Lines 413-425	Expand the discussion of the gender imbalance in your study's sample and explore how this might affect the validity and applicability of the study results to the broader SLE population.
10	Lines 199-208	Enhance the cultural and linguistic validation of the survey tools used, ensuring they are appropriate for the Mexican context and diverse ethnic groups within the country.
