# Supplementary material for: Quality of life disparities among Mexican people with systemic lupus erythematosus
Source: PLOS Digit Health. 2025 Jan 23;4(1):e0000706. doi: 10.1371/journal.pdig.0000706 (PMC11756751; doi:10.1371/journal.pdig.0000706)
Supplement: Attachment — Submitted filename: Response_to_reviewers.pdf [file pdig.0000706.s004.pdf]

Journal Requirements:

1. Please amend your detailed online Financial Disclosure statement. This is published with the article. It must therefore be completed in full sentences and contain the exact wording you wish to be published.
a) State the initials, alongside each funding source, of each author to receive each grant. For example: "This work was supported by the National Institutes of Health (####### to AM; ###### to CJ) and the National Science Foundation (###### to AM)."

b) State what role the funders took in the study. If the funders had no role in your study, please state: "The funders had no role in study design, data collection and analysis, decision to publish, or preparation of the manuscript."
We added the statement concerning the funders role in the study.

2. Please provide separate figure files in .tif or .eps format only and remove any figures embedded in your manuscript file. Please also ensure that all files are under our size limit of 10MB. You may leave the figure captions or legends in the manuscript.
For more information about how to convert your figure files please see our guidelines: https://journals.plos.org/digitalhealth/s/figures
As suggested in the guidelines, all figures were converted using the PACE app.
https://drive.google.com/drive/folders/17M5l4Yguc_TXFLddIEWQIeR5USahpdV_

3. We have noticed that you have cited supplementary material "Supplementary Figure 1" on page 21 in your manuscript. However, there is no corresponding file uploaded to the submission. Please upload it as separate file with the item type 'Supporting Information'.
Please ensure that the Supporting Information file has a legend listed in the manuscript after the references list.
We apologize for the distraction and omission about Supplementary Figure 1, we have added the file to the submission system.

4. We noticed that you used "data not shown" in the manuscript. We do not allow these references, as the PLOS data access policy requires that all data be either published with the manuscript or made available in a publicly accessible database. Please amend the supplementary material to include the referenced data or remove the references.
We appreciate the observation, the data is available on github corresponding to the data analysis made (*https://github.com/NeuroGenomicsMX/Lupus_RGMX_analysis*). Furthermore, we added a graph as supplementary figure 2, and we also added this information in the before ahead mentioned part:

"The validation dataset confirmed the importance (30% of the data); in addition, we ran a multivariate adaptive regression splines model, and the variable importance was corroborated, but in distinct order (see supplementary Figure 2)."

Additional Editor Comments (if provided):

The review process for your manuscript is complete. Based on the reviewers' comments, I concluded to accept this manuscript with further revision.
It is important that in your review you take into account all the reviewers' comments, especially in the methodological part and comments on the limitations of the study.
Once you have reviewed your manuscript, please follow the instructions for submitting it

Reviewers' comments:

Reviewer's Responses to Questions

Comments to the Author
1. Does this manuscript meet PLOS Digital Health’s publication criteria? Is the manuscript technically sound, and does the data support the conclusions? The manuscript must describe methodologically and ethically rigorous research with conclusions that are appropriately drawn based on the data presented.
Reviewer #1: Yes
Reviewer #2: Yes
----

2. Has the statistical analysis been performed appropriately and rigorously?
Reviewer #1: Yes
Reviewer #2: I don't know
----

3. Have the authors made all data underlying the findings in their manuscript fully available (please refer to the Data Availability Statement at the start of the manuscript PDF file)?
The PLOS Data policy requires authors to make all data underlying the findings described in their manuscript fully available without restriction, with rare exception. The data should be provided as part of the manuscript or its supporting information, or deposited to a public repository. For example, in addition to summary statistics, the data points behind means, medians and variance measures should be available. If there are restrictions on publicly sharing data—e.g. participant privacy or use of data from a third party—those must be specified.
Reviewer #1: Yes
Reviewer #2: Yes
----

4. Is the manuscript presented in an intelligible fashion and written in standard English?
PLOS Digital Health does not copyedit accepted manuscripts, so the language in submitted articles must be clear, correct, and unambiguous. Any typographical or grammatical errors should be corrected at revision, so please note any specific errors here.
Reviewer #1: Yes
Reviewer #2: Yes
----

5. Review Comments to the Author

Please use the space provided to explain your answers to the questions above. You may also include additional comments for the author, including concerns about dual publication, research ethics, or publication ethics. (Please upload your review as an attachment if it exceeds 20,000 characters)

Reviewer #1:

- In the introduction, add the citation regarding the prevalence of SLE. Where were the statistics obtained from?

We added two references regarding the worldwide prevalence of SLE:

  - Barber, M. R. W., Drenkard, C., Falasinnu, T., Hoi, A., Mak, A., Kow, N. Y., Svenungsson, E., Peterson, J., Clarke, A. E., & Ramsey-Goldman, R. (2021). Global epidemiology of systemic lupus erythematosus. Nature reviews. Rheumatology, 17(9), 515–532. https://doi.org/10.1038/s41584-021-00668-1
  - Barber, M. R. W., Falasinnu, T., Ramsey-Goldman, R., & Clarke, A. E. (2023). The global epidemiology of SLE: narrowing the knowledge gaps. *Rheumatology (Oxford, England)*, *62*(Suppl 1), i4–i9. https://doi.org/10.1093/rheumatology/keac610

Regarding the prevalence of SLE in México we added some specific data recovered from the work from Peláez-Ballestas:

”In Mexico, previous epidemiological studies across five regions (Mexico City, Nuevo León, Yucatán, Sinaloa, and Chihuahua) estimated an SLE prevalence of approximately 60 cases per 100,000 inhabitants, with a range of 40 to 90 cases per 100,000 (14).

  - Peláez-Ballestas, I., Sanin, L. H., Moreno-Montoya, J., Alvarez-Nemegyei, J., Burgos-Vargas, R., Garza-Elizondo, M., Rodríguez-Amado, J., Goycochea-Robles, M. V., Madariaga, M., Zamudio, J., Santana, N., Cardiel, M. H., & Grupo de Estudio Epidemiológico de Enfermedades Músculo Articulares (GEEMA) (2011). Epidemiology of the rheumatic diseases in Mexico. A study of 5 regions based on the COPCORD methodology. *The Journal of rheumatology. Supplement*, *86*, 3–8. https://doi.org/10.3899/jrheum.100951

- In the method section, add a list of definitions for the key terms used in the study.

At the end of the method section, we added Table 1, which describes the key features for the analysis, their units, and range.

- Regarding the parameters collected, add a table showing the main parameters and by how many participants were reported. Were there any missing values?

At the end of the method section, we added table 2, describing how many responses we collected for the most important features. We also show the count of missing values.

- The RMSE should be reported for both the calibration and test sets. The closer both values the more robust is the model.

RMSE is now reported for both the training and the validation dataset. We added the next text in the results section:

"The random forest model showed RMSE = 9.5 in the training dataset (70% of the data) and RMSE = 9.8 in the validation dataset (30%)."

- Comment on the generalisability of the findings.

Regarding the generalizability of our findings, we added the next paragraph in the discussion section.

"We consider our sample to be representative of the country, as the age range is wide, it includes patients covered by different, and covers different geographical regions, albeit with better representation from the region defined that comprehends Mexico City, the State of Mexico, Queretaro, Morelos, and Puebla. However, considering this region receives migration from all other states and concentrates a large proportion of specialized hospitals and medical services, the results can be cautiously extrapolated to the whole country. In addition, we observed an uneven representation of the different socioeconomic status, with almost 40% of our participants belonging to low economic status levels, which can limit the generalizability of our findings to higher socioeconomic levels."

- Could do with a language revision and proofreading.

We thank the reviewer for the suggestion, we have reviewed the draft of the manuscript.

Reviewer #2:

Dear Author,
Your manuscript on "Quality of life disparities among Mexican people with systemic lupus erythematosus" presents valuable insights into an important topic. However, several aspects of the manuscript need attention to enhance its quality and impact.

- Firstly, there is a need for more detailed explanations regarding the choice of parameters in your models, particularly the hyperparameters in the random forest model. This will help readers understand the rationale behind your modeling decisions and ensure transparency and reproducibility.

Regarding the choice of hyperparameters in the random forest model, we provided more details for the parameters and the search grid. We also detailed parameters for the confirmatory multivariate adaptive regression splines model.

"After verifying data consistency, we removed 221 unmatched registries, so from 942 individuals with SLE, only 721 were considered for the random forest model. We split 70% of the data to train the model and left 30% for validation. All 43 predictive variables were transformed into binary by the one-hot technique. Since we have noisy variables, we ran hyperparameters' optimization. Specifically, we trained the base model by running a full Cartesian grid search (all combinations of hyperparameters in the grid) with the following hyperparameters: number of trees = 10 times number of predictive variables; sampling with and without replacement;

resampling fraction: {0.4, 0.5, 0.6, 0.7, 0.8}; minimum node size: {1, 3, 5, 10, 20, 30, 40, 50}; mtry: {0.05, 0.15, 0.25, 0.33, 0.4, 0.6, 0.7, 0.8, 1}. In the following sentences, we explain the rationale behind these parameters.

To stabilize the error rate, we chose 430 trees (10 times the number of predictive variables). Sampling without replacement was explored due to the many unbalanced categories, which can yield less biased results (35). Reducing the resampling fraction from 0.8 to 0.4, it was possible to minimize tree correlation when a few variables dominated predictions. Increasing the minimum node size to 50 was recommended to manage noisy predictors (36), and mtry values were adjusted up to 1 to enhance performance with noisy data (37). Once the best parameters were identified, we re-ran the model with optimal parameters and 5000 trees to obtain stable error estimates. We calculated variable importance using the permutation approach. These importance values were validated on the validation dataset.

We used a multivariate adaptive regression splines model (cross-validated) to corroborate the random forest results and calculated marginal effects for the most influential variables (37,38). This model used a search grid with maximum degrees of interaction ranging from 1 to 3 and the number of terms retained in the final model ranging from 2 to 100, increasing by 5. The multivariate adaptive regression splines model was selected because it is non-parametric, allowing us to validate the random forest results, and it can detect non-linear interactions between predictive and outcome variables. This is important because we suspected that QoL in lupus might not have a linear relationship with some predictive variables."

- Secondly, the justification for using non-parametric tests and treating Likert scale data as numeric variables should be provided. Clarifying these methodological choices will strengthen the validity of your statistical analysis.

We have extended such justification:

"Note that all tests and models applied in the present study are non-parametric, and they can handle continuous and categorical data (28–31). However, given that our questionnaire design presented a Likert scale equally spaced, showing visual symmetry around the central point, and kept precisely the same range for all the items, we will be able to treat and interpret them as numeric variables on the interval scale (32, 33). Moreover, aggregated questionnaires (e.g, WHOQoL and NSE 2022) could be handled as continuous because of the central limit theorem (33, 34). This way, we gain statistical power, for example, in the permutation test."

- Additionally, the literature review would benefit from including more recent studies focusing on the Mexican population or similar contexts. Also, consider broadening the range of studies from different geographical regions to provide a more comprehensive overview of the topic.

We thank the reviewer for this suggestion. We've added some perspectives about QoL in people with SLE in the introduction of the manuscript.

"In this context, evaluating the quality of life (QoL) offers valuable insights into how lupus diagnosis affects individuals' lives. Numerous studies have identified the impact of ethnicity and sociodemographic factors, such as education level and economic status, on QoL perceptions among SLE patients. For instance, individuals of European ancestry have reported lower physical functioning QoL metrics

compared to those of non-European ancestries, while African American patients have reported lower psychosocial QoL components. Since QoL perception in lupus patients has been linked to treatment adherence and communication with healthcare providers, integrating QoL assessment into SLE management is crucial (16,17).

A recent study in Mexico reported lower QoL among people with rheumatologic diseases; specifically, for Mexican women with SLE, lower QoL has been observed when compared with non-SLE-affected controls; this lower QoL perception among people with SLE can also be observed when compared with other rheumatologic diseases such as rheumatoid arthritis (16,18)."

- Methodologically, it's essential to include information on the validation of the questionnaires used in the Mexican context.

Regarding the World Health Organization Quality of Life questionnaire we have added in the text:

"The QoL questionnaire employed the Spanish version of the World Health Organization QoL Questionnaire (WHOQOL-BREF), previously validated across various populations, including Latin Americans, and widely used in clinical populations in Mexico, including those with rheumatic diseases (21–24)."

We have included the references regarding the development and validation of the spanish version of the WHOQOL questionnaire (Lucas-Carrasco, 2012), as well as reference to its appliance to assess a clinical population in México with rheumatic diseases (Guaracha-Basáñez et al., 2022).

For the socioeconomic evaluation we included the reference to the review and validation of NSE rule 2022 by the Mexican Association of Market and Opinion Intelligence Agencies (AMAI). and we addressed it on the text:

"This algorithm is reviewed and updated every two years by AMAI based on the National Household Income and Expenditure Survey (ENIGH) conducted by the National Institute of Statistics and Geography (INEGI). The NSE 2022 rule is considered a standard criterion for socioeconomic classification in Mexico and has been validated using ENIGH data (26,27)."

- Further elaboration on the rationale behind choosing specific statistical tests and models will improve the clarity and rigor of your methodology section.

We added some lines to the sections in which every test and model were explained. Specifically, these lines explain in more detail the reasons why we choose each of them.

"We selected the permutation test because, among many comparative tests, it has the greatest statistical power (28)". Section 2.3.4 Statistical analysis. Page 9

"Second, a random forest model was applied to identify predictors of QoL. QoL was used as the outcome variable, and age, sex, years living with SLE, treatment, comorbidities, health provider, diagnostic criteria, diagnosis delay, and socioeconomic status were used as predictive variables. The random forest method was selected for its good predictive performance, the relatively few hyperparameters required, and its suitability for optimizing through a full grid search without requiring

extensive computational resources. It also handles noisy and influential predictive features that do not meet the assumptions of traditional linear regression models." Section 2.3.4 Statistical analysis. Page 10

"We used a multivariate adaptive regression splines model (cross-validated) to corroborate the random forest results and calculated marginal effects for the most influential variables (37,38). This model used a search grid with maximum degrees of interaction ranging from 1 to 3 and the number of terms retained in the final model ranging from 2 to 100, increasing by 5. The multivariate adaptive regression splines model was selected because it is non-parametric, allowing us to validate the random forest results, and it can detect non-linear interactions between predictive and outcome variables. This is important because we suspected that QoL in lupus might not have a linear relationship with some predictive variables." Section 2.3.4 Statistical analysis. Page 11

"The permutation test was selected for its statistical power, and FDR correction was applied because we ran six comparisons among the groups". Section 2.3.4 Statistical analysis. Page 12

- In the discussion section, thoroughly discussing the limitations of the study, including potential biases in self-reported data and the representativeness of the sample, is crucial. Furthermore, providing specific recommendations for policy or clinical practice based on your findings and linking them more explicitly to the existing literature will enhance the relevance and impact of your study.

We expanded and explained in more detail the limitations of our study. We also adapted the discussion, depicting the representativeness of the sample and generalization of findings. Furthermore, we also included specific recommendations for clinicians as well as for health policymakers.

"Limitations of this study include low participation in some geographical regions, accessibility to the platform, the self-reported appliance of surveys, uneven representation of all the socioeconomic status, and the lack of standard measures to assess lupus activity, such as the Systemic Lupus Erythematosus Disease Activity Index (SLEDAI) which can be only determined by rheumatologists.

We consider our sample to be representative of the country, as the age range is wide, it includes patients covered by different, and covers different geographical regions, albeit with better representation from the region defined that comprehends Mexico City, the State of Mexico, Queretaro, Morelos, and Puebla. However, considering this region receives migration from all other states and concentrates a large proportion of specialized hospitals and medical services, the results can be cautiously extrapolated to the whole country. In addition, we observed an uneven representation of the different socioeconomic status, with almost 40% of our participants belonging to low economic status levels, which can limit the generalizability of our findings to higher socioeconomic levels.

According to our data, patients show a better quality of life perception when diagnosed within a year from the first symptoms. Delays in diagnosis are critical factors to consider, 23% of our participants reported that their diagnoses were delayed more than five years, which can contribute to medium- or long-term complications. For example, other studies reported that delay in diagnosis increases the risk of disease-related damage and higher disease activity. In contrast, sooner-diagnosed people show a lower flare rate (51–53). Our findings also highlight that people at lower socioeconomic levels showed lower quality of life; further analysis will explore the potential impact this can have on the patients, since other studies associated lower socioeconomic status with higher organ damage (54). The Mexican College for Rheumatology has ongoing programs to train general practitioners from public health providers to identify potential lupus cases and speed up referring them to a specialist for assessment. These efforts should continue to be supported.

This work is the first step towards understanding the quality of life in Mexican people with SLE and the potential variables contributing to it. As previously mentioned, quality of life evaluation represents a valuable tool to assess the effect a chronic disease, such as SLE, has on a patient's life. The interdisciplinary and synergistic work between clinicians, researchers, and people with SLE through implementing the LupusRGMX platform has established a pioneering approach that provides a valuable source of data about QoL in Mexican people with SLE."

Overall, addressing these comments will strengthen your manuscript and ensure it makes a meaningful contribution to the field.
