# Supplementary material for: Quality of life disparities among Mexican people with systemic lupus erythematosus
Source: PLOS Digit Health. 2025 Jan 23;4(1):e0000706. doi: 10.1371/journal.pdig.0000706 (PMC11756751; doi:10.1371/journal.pdig.0000706)
Supplement: Attachment — Submitted filename: review.pdf [file pdig.0000706.s005.pdf]

1. Introduction

- **Current text (line 122-125)**: "Disparities have been reported among people with SLE, not only regarding the prevalence, but also in outcomes associated with their clinical and psychological status; higher prevalence, disease activity and mortality rates have been reported among Latin-American, admixed North-American, African, and Native-American populations."

Recommendation: Consider rephrasing for clarity and flow. Example:

  - "Disparities in SLE prevalence and outcomes have been observed across different populations, with higher disease activity and mortality rates among Latin-American, African-American, and Native-American populations, compared to their European counterparts."

- **Current text (line 130-133)**: "Latin-America exhibits great inequalities in economy, policies, employment, health access and violence; the impact that these inequalities may have on people with SLE in Latin-American countries has been scarcely explored."

Recommendation: Provide citations for the stated disparities and briefly explain the relevance to SLE outcomes. Example:

  - "Latin America faces significant inequalities in economic, healthcare access, and social stability, all of which can contribute to poorer health outcomes for SLE patients. However, these socioeconomic determinants in Latin-American SLE populations remain underexplored (add citations)."

2. Methodology

- **Current text (line 214)**: "All curated, preprocessing, and data analyses were developed and implemented in R 4.3.1."

Recommendation: Add details on handling missing data. Example:

  - "All curated, preprocessing, and data analyses were developed and implemented in R 4.3.1. Missing data were handled through imputation techniques (if applicable) or by excluding cases with incomplete data (if applicable)."

- **Current text (lines 243-250)**: "Since we have noisy variables, we trained the base model running a search grid with the following hyperparameters: number of trees = 10 times number of predictive variables; sampling with and without replacement; resampling fraction: {0.4, 0.5, 0.6, 0.7, 0.8; minimum node size: {1, 3, 5, 10, 20, 30, 40, 50; mtry: {0.05, 0.15, 0.25, 0.33, 0.4, 0.6, 0.7, 0.8, 1} times number of predictive variables. All predictive variables were transformed to binary by one hot technique."

Recommendation: Explain why these hyperparameters were selected. Example:

- “Since noisy variables were present, we conducted hyperparameter tuning by using a grid search approach, optimizing parameters such as the number of trees, resampling fraction, and minimum node size to maximize model performance. These parameters were chosen to balance predictive accuracy while controlling for overfitting due to noise.”

- **Current text (lines 272-278)**: “However, given that our questionnaire design presented the Likert scale equally spaced, showing visual symmetry around the central point, and kept precisely the same range for all the items, we also would be able to treat and interpret them as numeric variables into the interval scale.”

Recommendation: Justify treating Likert scale data as interval. Example:

- “Although Likert scale data are ordinal, the visual symmetry of the response options and equal spacing between points in our questionnaire justify treating them as interval data, which enhances the statistical power of our analysis.”

3. Results

- **Current text (line 280)**: “Regarding QoL, we found significant differences between the SLE-group and both healthy control groups for the overall score and all domains of QoL.”

Recommendation: Make this statement more precise by including statistical details upfront. Example:

- “Significant differences in QoL were observed between the SLE group and both healthy control groups (p-values < 0.0001) across the overall score and all QoL domains (Figure 1).”

- **Current text (lines 301-306)**: “Concerning the random forest model, it showed a RMSE = 9.5 in the training dataset (70% of data). We found that socioeconomic status, delay in diagnostic (or diagnostic lag), and corticosteroid consumption are the variables with the greater impacts on QoL.”

Recommendation: Report RMSE for both training and validation datasets. Example:

- “The random forest model achieved an RMSE of 9.5 in the training dataset (70% of the data) and 9.8 in the validation dataset (30%). The most impactful variables on QoL were socioeconomic status, diagnostic delay, and corticosteroid consumption, as determined by variable importance analysis.”

- **Current text (lines 309-311)**: “Higher socioeconomic status is associated with better QoL (Figure 2A), whereas greater diagnostic lag (Figure 2B) and corticosteroid consumption (Figure 2C) are associated with lower QoL.”

Recommendation: Expand this section by explaining why these variables are significant. Example:

- "As shown in Figure 2, higher socioeconomic status correlated with better QoL scores, likely due to better access to healthcare resources. Conversely, longer diagnostic lag and corticosteroid use were associated with reduced QoL, reflecting the negative impact of delayed treatment initiation and the adverse side effects of long-term corticosteroid therapy."

4. Discussion

- **Current text (lines 375-378)**: "Higher socioeconomic status is associated with better QoL, which is in line with previous studies that have proposed that health care system structure may not be equally accessible for all."

Recommendation: Expand the explanation of health access disparity. Example:

- "Higher socioeconomic status was positively associated with better QoL, supporting findings from other studies that highlight the inequities in healthcare access. Wealthier individuals often have access to private healthcare, shorter wait times, and more specialized care, while those from lower socioeconomic strata face barriers such as overburdened public systems and limited specialist availability."

- **Current text (lines 413-416)**: "Limitations of this study include low participation in some areas, accessibility to the platform, the self-reported nature of surveys, lack of representation of some socioeconomic status and standard measures to assess SLE."

Recommendation: Be more specific about the limitation and how to address it. Example:

- "Limitations of this study include lower participation from certain geographic regions, which may limit the generalizability of findings. Additionally, the reliance on self-reported surveys could introduce bias, particularly in health status and QoL measurements. Future studies should aim to include objective clinical assessments and ensure more representative sampling across socioeconomic groups."

5. Language and Proofreading

- **Line 292**: The word "punctuated" is used when referring to scores.

Recommendation: Use "scored" instead. Example:

- "SLE patients scored 2.13 points lower on social-relations than the control group."

- **Line 421**: The phrase "this work settles a first step" can be made clearer.

Recommendation: Rephrase to improve clarity. Example:

    - "This study represents an important first step toward understanding the impact of SLE on QoL in Mexican populations."

Summary of Changes:

- Clarify methodology, particularly regarding the random forest model and treatment of Likert scale data.
- Improve the explanation of limitations, especially regarding missing data and the generalizability of findings.
- Strengthen the introduction by discussing the gap in the literature earlier and providing better citation of statistics.
- Revise language for fluency and consistency across the manuscript, ensuring technical terms are used correctly.
